# Seasonal and spatial heterogeneities in host and vector abundances impact the spatiotemporal spread of bluetongue

**DOI:** 10.1186/1297-9716-44-44

**Published:** 2013-06-19

**Authors:** Maud VP Charron, Georgette Kluiters, Michel Langlais, Henri Seegers, Matthew Baylis, Pauline Ezanno

**Affiliations:** 1 INRA, UMR1300 Biologie, Epidémiologie et Analyse de Risques en santé animale, CS 40706, F-44307 Nantes, France; 2 LUNAM Université, Oniris, Ecole nationale vétérinaire, agroalimentaire et de l’alimentation Nantes-Atlantique, UMR BioEpAR, F-44307 Nantes, France; 3 Institut de Mathématiques de Bordeaux, UMR 5251, Université de Bordeaux, F33076 Bordeaux, France; 4 CNRS, IMB, UMR 5251, F-33400 Talence, France; 5 Institute of Infection and Global Health, Liverpool University Climate and Infectious Diseases of Animals (Lucinda) Group, Leahurst Campus, University of Liverpool, Neston CH64 7TE, UK

## Abstract

Bluetongue (BT) can cause severe livestock losses and large direct and indirect costs for farmers. To propose targeted control strategies as alternative to massive vaccination, there is a need to better understand how BT virus spread in space and time according to local characteristics of host and vector populations. Our objective was to assess, using a modelling approach, how spatiotemporal heterogeneities in abundance and distribution of hosts and vectors impact the occurrence and amplitude of local and regional BT epidemics. We built a reaction–diffusion model accounting for the seasonality in vector abundance and the active dispersal of vectors. Because of the scale chosen, and movement restrictions imposed during epidemics, host movements and wind-induced passive vector movements were neglected. Four levels of complexity were addressed using a theoretical approach, from a homogeneous to a heterogeneous environment in abundance and distribution of hosts and vectors. These scenarios were illustrated using data on abundance and distribution of hosts and vectors in a real geographical area. We have shown that local epidemics can occur earlier and be larger in scale far from the primary case rather than close to it. Moreover, spatial heterogeneities in hosts and vectors delay the epidemic peak and decrease the infection prevalence. The results obtained on a real area confirmed those obtained on a theoretical domain. Although developed to represent BTV spatiotemporal spread, our model can be used to study other vector-borne diseases of animals with a local to regional spread by vector diffusion.

## Introduction

There is significant concern regarding the resurgence or emergence of vector-borne diseases of animals with serious consequences for animal health and economics [1-3]. Climate change and socio-economic change are both believed to contribute to the emergence and spread of such diseases [4]. A recent example is the unexpected introduction of the serotype 8 of the bluetongue virus (BTV) in northern Europe in 2006. Bluetongue is a non-contagious vector-borne disease affecting domestic and wild ruminants with high direct and indirect economic consequences [5,6]. It spread for three years with an annual epidemic peak followed by the disappearance of clinical cases.

A better understanding of the temporal and spatial spread of BTV has direct consequences for the disease prevention and control. The recent incursion of BTV in Europe has been controlled using a massive vaccination. To propose alternative to such a massive strategy if BTV incursions were to occur, we need to better identify where and when targeted strategies should be implemented. Therefore, the occurrence and amplitude of both local (a few km^2^) and regional epidemics should be more precisely predict.

The spatiotemporal heterogeneity in abundance and distribution of hosts and vectors generally has a strong impact on pathogen spread and persistence [7,8]. In a seasonal environment such as in Europe, bluetongue is characterized by strong seasonal variations in incidence related to the seasonality of the vector population [9,10], whose lifecycle largely depends on environmental factors, such as humidity and temperature. As a result, clinical cases almost disappear during the unfavourable season for the vector. In addition to the temporal heterogeneity in vector abundance, the heterogeneity in the spatial distribution of vectors and hosts may also impact bluetongue spread [11,12]. Such heterogeneities in vector and host abundance and distribution can differ between geographic areas. In livestock populations, they relate to the landscape structure as well as to farming practices as animal populations are managed by farmers.

Mathematical modelling is a relevant approach for investigating the spread of vector-borne diseases [8,13-16]. As hosts and vectors are mobile entities, a spatial component in vector-borne disease models should be taken into account to better consider the evolution of the biological system [17-19]. This spatial component is not only due to space structuring in terms of density and location of host and vector populations, but also to host and vector movements over space. Different methods of various levels of complexity exist to include this spatial component in epidemiological models. To study the spread of vector-borne diseases, spatially explicit models are generally preferred [13,20]. They permit to take into account both vector active and passive movements and host movements that occur at different scales. Moreover, such models have been used also to describe the velocity of travelling waves of epidemics [8,14,16].

Such a modelling approach has been used to represent the spatiotemporal BTV spread in specific areas [21-24]. If the published models took into account the heterogeneity in distribution and abundance of livestock populations, they tend to assume uniform densities of vectors (i.e. the same number per farm) or uniform host: vector ratio (i.e. more midges on bigger farms) [21-24]. Recently, it has been shown that these are probably unrealistic [25]. Therefore, models that take full account of vector heterogeneity both in space and time have to be developed.

Our objective was to assess, using a modelling approach, how spatiotemporal heterogeneities in abundance and distribution of hosts and vectors impact the occurrence and amplitude of local and regional BT epidemics. We first studied different hypothetical scenarios of spatial heterogeneity in host and vector populations, and then illustrated the theoretical results in a real geographic area.

## Material and methods

### Model description

**Figure 1:**
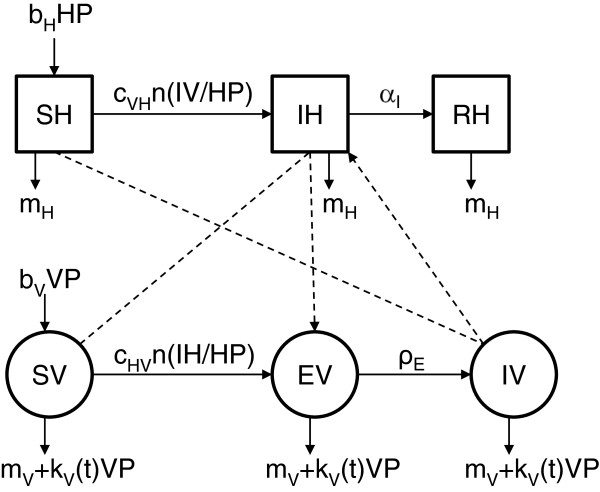
**Conceptual model of BTV8 spread.** Flow diagram describing the model used for BTV8 spread in midge and cattle populations. Squares represent the health states of hosts (H), circle those of vectors (V), with S for susceptible, E for latent, I for infectious, R for recovered. The descriptions, values and sources of all parameters in the epidemiological model are found in Table 1.

Three actors are necessary for bluetongue spread: the virus (here BTV8), the vector (a midge, here *Culicoides*), and the host (a ruminant, here cattle). As the virus is not excreted, we have assumed all transmission is vectorial. The developed model is based on a more complex model of the seasonal temporal spread of bluetongue in cattle [26]. This model has been simplified and made spatial. Here, the vertical transmission (in utero) has not been taken into account, as this hypothesis has not been shown to influence the infection [26]. The temporal dynamics of the vector population has been modified using a more flexible function. We used a standard compartment model (Figure 1) to describe the transmission of a pathogen between a vertebrate host population (*HP*) and a vector population (*VP*). The parameters of this model are defined in Table 1. The host population (*HP*) is divided into three health states: susceptible (*SH*), infectious (*IH*), and immune (*RH*). It is assumed to remain constant: the entry rate (*b*_*H*_) compensates the exit rate (*m*_*H*_). The vector population (*VP*) is divided into three health states: susceptible (*SV*), exposed (*EV*), and infectious (*IV*). For vectors, a latency period (1/*ρ*_*E*_; Table 1) was taken into account as it is of the same order as life expectancy. At the disease-free state and with seasonality, the vector population was assumed to have a logistic growth with *K(t) =* (*b*_*V*_*-m*_*V*_)*/k*_*V*_*(t)* (Table 1), the carrying capacity of the environment depending on the vector fertility (*b*_*V*_), mortality (*m*_*V*_) and density-dependant mortality (*k*_*V*_) rates. In periods favourable for vectors, the *K* function is a sinusoidal function with a maximum *h*. In unfavourable periods, the *K* function is constant and equal to *Nb*. The vectorial transmission takes place when an infectious vector (*IV*) bites a susceptible host (*SH*) which becomes infectious (*IH*), or when a susceptible vector (*SV*) bites an infectious host (*IH*) and then becomes exposed (*EV*). Incidence functions were frequency dependant for hosts and vectors. The mean host viremia duration (i.e. the time spent in *IH*) was 1/α_I_. With recovery, animals move from state *IH* to *RH*.

**Table 1:** Parameters of the model of BTV8 spread in midge and cattle populations

**Host parameters**	**Description**	**Value**	**References**
*b*_*H*_	Birth rate (per day)	6,94.10^-4^	a
*m*_*H*_	Exit rate (mortality, selling, culling) (per day)	*b*_*H*_	
*1/α*_*I*_	Duration of viremia (days)	60	[43]
*c*_*VH*_	Probability of transmission from vector to host	0.92	[44,45]
**Vector parameters**	**Description**	**Value**	**References**
*c*_*HV*_	Probability of transmission from host to vector	0.15	[46,47]
*n*	Biting rate (per day)	0.25	[45]
*b*_*V*_	Fertility rate (per day)	6.1	[45]
*m*_*V*_	Mortality rate (per day)	1/21	[34,45]
*K(t)*	Carrying capacity	*	
*k*_*V*_	Density-dependence mortality rate (per day)	*(b*_*V*_*-m*_*V*_*)/K(t)*	
*h*	Maximum of *K(t)*	variable	
*d*	Duration of favourable period (days)	243	
*Nb*	Number of vectors during the unfavourable period	100	
*1/ρ*_*E*_	Duration of extrinsic incubation period (days)	10	[4,45]
*D*	Diffusion coefficient (km^2^/day)	1,25.10^-2^	

* K(t) = 1_*[1 ; d]*_(t)*[ h*sin(|π*(365-t)/d|)] + 1*_*[d+1;365]*_(t)*Nb.

a: agricultural statistics.

Let Ω be the square spatial domain. *X* = *X* (*x*, *y, t*) represents time dependant population densities in (*x*, *y*) ∈ Ω. During the epidemic, host movements are controlled, therefore the spatial spread of the epidemic is due to vector movements rather than host movements. Moreover, we focus on a local to regional scale, and therefore assume that the spatial spread of BTV8 is exclusively due to local movements of vectors. BTV8 having no detrimental impact on vectors, thereby the diffusion process is similar whatever the health state. Therefore, the diffusion process follows the first Fick’s law:

$$\vec{q}\left(x,y,t\right)=-D\left(x,y\right).\nabla \mathrm{SV}\left(x,y,t\right),$$

where $\vec{q}\left(x,y,t\right)$ is the diffusion flux of the vector population and *D(x,y)*, the positive diffusion matrix. Appling the conservation law (i.e*.* the variation of the amount of species in a volume is equal to the balance of entering and outgoing flux), we obtain the second Fick’s law: $\frac{\partial \mathrm{SV}\left(x,y,t\right)}{\partial t}+\mathrm{div}\vec{q}\left(x,y,t\right)=0.$ Combining these two laws, we obtain: $\frac{\partial \mathrm{SV}\left(x,y,t\right)}{\partial t}=-\mathrm{div}\vec{q}\left(x,y,t\right)=-\mathrm{div}\left(-D\left(x,y\right).\nabla \mathrm{SV}\left(x,y,t\right)\right)$We consider that the dispersion of vectors is homogeneous in space; therefore *D(x,y)* = *D*. Then, $\frac{\partial \mathrm{SV}\left(x,y,t\right)}{\partial t}=D\;\mathrm{div}\left(\nabla \mathrm{SV}\left(x,y,t\right)\right)=\mathrm{D\Delta SV}\left(x,y,t\right)$.

By adding the reaction term, we obtain the following system of equations (Eq. 1) describing the spatiotemporal spread of the BTV8, for (*x*, *y*)∈ Ω and *t* > 0:

(1) $$\left\{\begin{array}{l} \frac{\partial \mathrm{SH}}{\partial t}=-c_{\mathrm{VH}}n\frac{\mathrm{IV}}{\mathrm{HP}}\mathrm{SH}+b_{H}\mathrm{HP}-m_{H}\mathrm{SH}\\ \frac{\partial \mathrm{IH}}{\partial t}=c_{\mathrm{VH}}n\frac{\mathrm{IV}}{\mathrm{HP}}\mathrm{SH}-\left(\alpha_{I}+m_{H}\right)\mathrm{IH}\\ \frac{\partial \mathrm{RH}}{\partial t}=\alpha_{I}\mathrm{IH}-m_{H}\mathrm{RH}\;\\ \frac{\partial \mathrm{SV}}{\partial t}=\mathrm{D\Delta SV}-c_{\mathrm{HV}}n\frac{\mathrm{IH}}{\mathrm{HP}}\mathrm{SV}-\left(m_{V}+k_{V}\mathrm{VP}\right)\mathrm{SV}+b_{V}\mathrm{VP}\\ \frac{\partial \mathrm{EV}}{\partial t}=\mathrm{D\Delta EV}+c_{\mathrm{HV}}n\frac{\mathrm{IH}}{\mathrm{HP}}\mathrm{SV}-\left(m_{V}+k_{V}\mathrm{VP}\right)\mathrm{EV}-\rho_{E}\mathrm{EV}\\ \frac{\partial \mathrm{IV}}{\partial t}=\mathrm{D\Delta IV}+\rho_{E}\mathrm{EV}-\left(m_{V}+k_{V}\mathrm{VP}\right)\mathrm{IV} \end{array}\right)$$

The initial time corresponds to the first day of the favourable season for the vector population. The spatial domain (Ω) is discretized into cells; each cell of surface area of 1 km^2^. Initially, all hosts and vectors are susceptible, except the primary case which corresponds to an infected host, placed in the centre of the grid, in a cell where there are hosts. We set non-negative initial conditions (Eq. 2). The flow of individuals across the domain boundary is assumed to be zero, i.e. we do not consider immigration or emigration of individuals and set Neumann boundary conditions (Eq. 2).

(2) $$\left\{\begin{array}{l} \mathrm{SH}\left(x,y,0\right)=SH^{0}(x,y),\mathrm{SV}(x,y,0)=SV^{0}(x,y)\\ \mathrm{IH}\left(x,y,0\right)=0\forall (x,y)\neq (21,21);\mathrm{IH}(21,21,0)=1\;\\ \mathrm{RH}\left(x,y,0\right)=\mathrm{EV}(x,y,0)=\mathrm{IV}(x,y,0)=0\;\\ \frac{\partial \mathrm{SH}}{\partial n}=\frac{\partial \mathrm{IH}}{\partial n}=\frac{\partial \mathrm{RH}}{\partial n}=\frac{\partial \mathrm{SV}}{\partial n}=\frac{\partial \mathrm{EV}}{\partial n}=\frac{\partial \mathrm{IV}}{\partial n}=0,\forall \left(x,y\right)\in \partial \Omega ,t>0 \end{array}\right)\;$$

To discretize the problem (Eq. 1 and 2) we used the finite difference method in space, and we converted the continuous time model into a discrete time one by using the semi-implicit Euler method, that we implemented in Scilab 5.1.

### Hypotheses of spatial heterogeneities in hosts and vectors

Assumptions are described by increasing level of complexity in Table 2. A first reference hypothesis (H1) considers a homogeneous spatial distribution and abundance in hosts and vectors. Four scenarios were studied for four maxima of the carrying capacity in vectors (*h1* to *h4*). From this assumption, four levels of heterogeneity were analyzed and compared.

**Table 2:** Hypotheses of spatial heterogeneities in abundance and distribution of hosts and vectors

	**H**	**Host**	**Vectors**	**Results figure**
**Homogeneous in hosts and vectors (4 Scenarios)**	H1	500 S/C 1 I in central cell Total number of hosts = 840 501	100 S/C *h1*=10^6^, or *h2*=10^7^ or *h3*=10^8^, or *h4*= 10^9^	5
**Heterogeneous in hosts Homogeneous in vectors (16 scenarios)**	H2	Total number of hosts S≈ 840 501 Four densities of occupied cells (Figure 2): 90% OC: 554 S/OC75% OC: 666 S/OC50% OC: 1000 S/OC25% OC: 2025 S/OC 1 I in central cell	H1	6 and 7
**Heterogeneous in vectors Homogeneous in hosts (2 scenarios)**	H3	H1	100 S/C Grid divided into four sub-areas of different maximum carrying capacities (*h1, h2, h3* and *h4*) (Figure 3a)	8
	H4		100 S/C 25% C: *h1*, 25% C: *h2*, 25% C: *h3*, 25% C: *h4* (Figure 3b)	9 and 10
**Heterogeneous in hosts and vectors (6 scenarios)**	H5	Crossing hypotheses: H2-H4		10 and 11
	H6	Real area (Figure 4b)	Real area (Figure 4a) Multiplication of the number of trapped vectors by 100 or 1000	12

We set *H* hypothesis, *C* cell, *OC* occupied cell. *S* susceptible individual, *I* infected individual.

**Figure 2:**
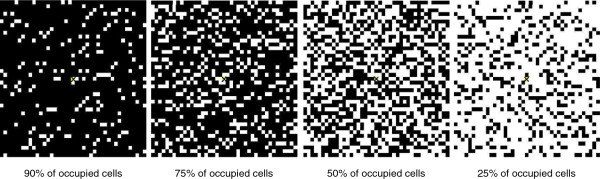
**Spatial distribution of cells occupied by hosts for four density levels (hypotheses H2 and H5).** Black cells correspond to occupied cells; white cells correspond to empty cells; the yellow and crossed cell corresponds to the cell wherein the primary case occurs.

First, a heterogeneous distribution of hosts was considered (H2), vectors being homogeneously distributed. We kept the total number of hosts on the grid constant. We tested five densities of occupied cells from 25% to 100%, where H1 is 100% of cells occupied (Figure 2). For each density, we randomly drew the occupied cells among the 1681 grid cells. For each scenario, three draws of occupied cells were compared. Results were identical whatever the distribution; therefore it did not influence the spatiotemporal spread of the virus on the grid. Thereafter, only one distribution was kept (Figure 2).

**Figure 3:**
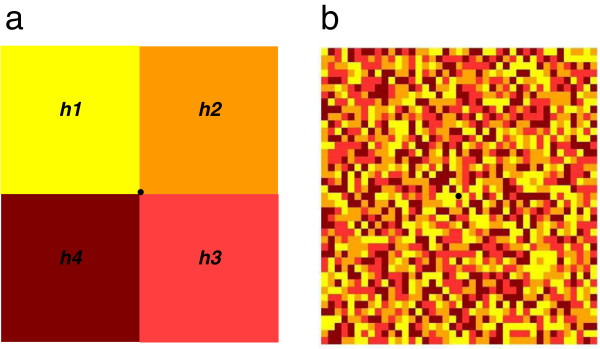
**Spatial distribution of the four maximum carrying capacities in vectors. a**) Hypothesis H3, **b**) Hypotheses H4 and H5. Yellow cells correspond to cells having a maximum carrying capacity in vectors given by *h1* = *10*^*6*^_*,*_ orange cells correspond to cells having a maximum carrying capacity in vectors given by *h2* = *10*^*7*^_*,*_ red cells correspond to cells having a maximum carrying capacity in vectors given by *h3* = *10*^*8*^, brown cells correspond to cells having a maximum carrying capacity in vectors given by *h4* = *10*^*9*^. The black cell corresponds to the cell wherein the primary case occurs.

Secondly, a heterogeneous distribution of vectors was considered (H3 and H4), hosts being homogeneously distributed. In H3, the domain (Ω) consisted of four sub-areas, each having a different maximum of the carrying capacity in vectors (*h1* to *h4*, Figure 3a). In H4, we considered a random equiprobable distribution of the same four maxima of the carrying capacity in vectors on the grid (Figure 3b).

Thirdly, we considered a heterogeneous distribution of hosts and vectors simultaneously (H5). This hypothesis crosses previous hypotheses H2 and H4.

Fourth, a last hypothesis (H6), based on real data, served to illustrate this theoretical work, in particular hypothesis H5.

### Data

**Figure 4:**
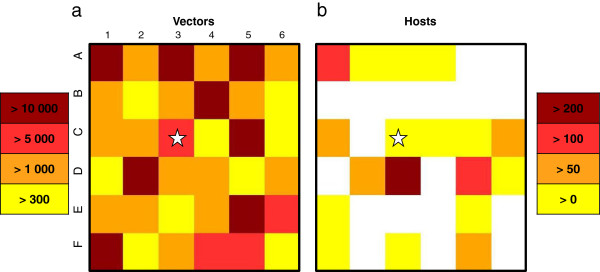
**Vector (a) and host (b) spatial distribution in the real area (in the Welsh province of Bala).** From yellow to brown, increasing numbers of individuals (white: empty cells); the cell with the star corresponds to the cell wherein the primary case occurs.

The *Culicoides* trap catches used for modelling were collected in the Welsh province of Bala, situated in Snowdonia National Park (for full methods, see [25]). The trapping farms were selected using a 6 × 6 km grid, whereby one farm was selected from each grid square (36 in total, Figure 4). Each farm was sampled for three nights using Onderstepoort-type down draught black light traps positioned as close to livestock as possible. Large collections were sub-sampled [27] and only females were considered in the analyses as males do not take blood meals or, consequently, transmit disease between vertebrates. The maximum trap-catch of *Culicoides* per farm, out of the three trapping nights, was used for modelling purposes (Figure 4a). Due to the nature of the terrain, two squares contained no properties. *Culicoides* counts for these grid squares were estimated using the models of [25]. The vector abundance is difficult to quantify. Therefore, we considered two scenarios, one multiplying the number of vectors per cell by 100, and another by 1000. The numbers of cattle per farm were recorded on questionnaires during data collection (Figure 4b). For farms with unknown numbers of cattle, values were interpolated using the known values of farms in adjacent grid squares.

### Outputs

The date and the prevalence at the epidemic peak in each cell were analysed, as well as the total prevalence on the grid over time. Thereafter, these three outputs are respectively named peak date, local prevalence and total prevalence. The peak dates were compared among the cells located on the four lines between the central cell (the half-diagonal), i.e. the cell of virus introduction, and the corners of the grid. This enabled us to numerically calculate an effective speed of the virus spread. We calibrated the diffusion coefficient (*D*) and the initial conditions (*SH00*^*0*^ and *SV*^*0*^) to have an effective speed of the virus spread similar to the estimated velocity by Pioz et al. [28], for hypothesis H1 and a maximum of the carrying capacity in vectors equals to 10^7^. The theoretical grid is a 41 × 41 km square, each half-diagonal measuring about 29 km. For hypotheses H3 to H6, peak dates and local prevalences were studied for comparable cells, i.e. equidistant and having the same maximum of the carrying capacity in vectors.

## Results

### Homogeneous in abundance and distribution of hosts and vectors (H1)

**Figure 5:**
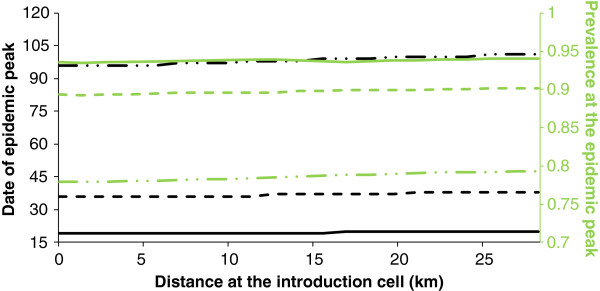
**Peak date and prevalence as a function of the distance from the primary case.** When hypothesis H1 holds. Black line corresponds to the peak date, green line corresponds to the prevalence. Solid lines correspond to a maximum carrying capacity in vectors given by *h4* = *10*^*9*^, dashed lines correspond to a maximum carrying capacity in vectors given by *h3* = *10*^*8*^, dashed-dotted lines correspond to a maximum carrying capacity in vectors given by *h2* = *10*^*7*^.

**Figure 6:**
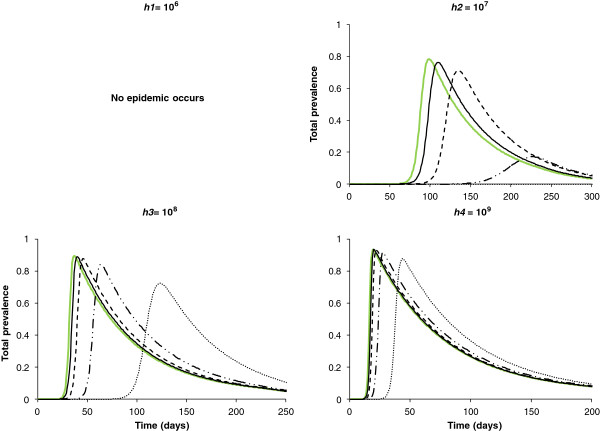
**Total prevalence over time.** In green when hypothesis H1 holds, in black when hypothesis H2 holds. Solid lines correspond to the case where 90% of cells are occupied, dashed lines correspond to the case where 75% of cells are occupied, in dashed-dotted lines correspond to the case where 50% of cells are occupied and dotted lines correspond to the case where 25% of cells are occupied.

For the lowest maximum of carrying capacity in vectors (*h1* = 10^6^), there is no epidemic. For the other values tested, there is an epidemic peak in all grid cells. A larger maximum of carrying capacity in vectors leads to an earlier peak and a faster speed of virus spread (Figure 5). For *h2* = 10^7^, five days are necessary for the virus to cover the half-diagonal from the cell of the virus introduction, with the epidemic peak occurring in the central cell and in the cells at the end of the half-diagonals at 96 and 101 days respectively after virus introduction. As expected in such a homogeneous environment, results are identical for the four half-diagonals. For *h3* = 10^8^ and *h4* = 10^9^, only two and one day, respectively, are necessary for the virus to cover the half-diagonals, the epidemic peak occurring 36 days (19 days, respectively) after virus introduction. A larger maximum of carrying capacity in vectors leads to larger local prevalences (Figure 5). Local prevalences are almost constant over different cells of the grid and are worth 78%, 90% and 94% for *h2*, *h3* and *h4* respectively. The total prevalence on the grid over time confirms these results. A larger maximum of carrying capacity in vectors leads to an earlier and larger epidemic peak (Figure 6).

### Heterogeneous in abundance and distribution of hosts and homogeneous in abundance and distribution of vectors (H2)

**Figure 7:**
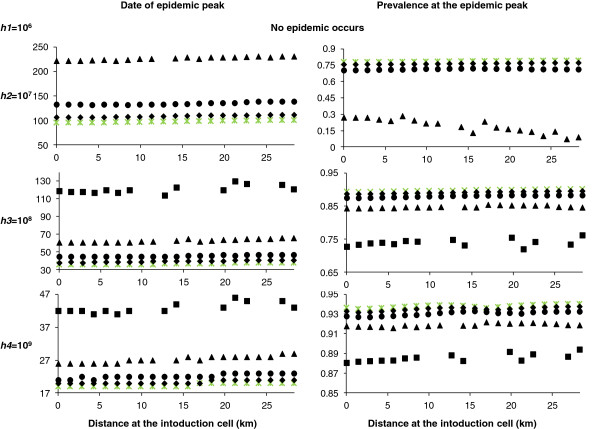
**Peak date and prevalence as a function of the distance from the primary case.** When hypothesis H2 holds. Green star correspond to the case where 100% of cells are occupied, lozenge correspond to the case where 90% of cells are occupied, circle correspond to the case where 75% of cells are occupied, triangle correspond to the case where 50% of cells are occupied and square correspond to the case where 25% of cells are occupied.

For all maxima of carrying capacity in vectors (except *h1* whatever the density of occupied cells and *h2* for a density of occupied cells equal to 25%, for which there is no epidemic) a smaller density of occupied cells leads to a later epidemic peak and a smaller local prevalence in all grid cells. For example, for a density of occupied cells equal to 75% and a maximum carrying capacity of *h2*, the epidemic peak was 37 days later than in H1, occurring at day 133 in the central cell and at day 139 in the cell at the end of the half-diagonal. Indeed, we kept the total number of hosts on the grid constant, so the number of hosts per occupied cell increases when the density of occupied cells decreases. As incidence functions are frequency dependent for hosts and vectors (Eq. 1), this increase in the number of hosts per occupied cell has little effect on the frequency of infection. However, the number of unoccupied cells increases, and therefore the virus transmission slows. The local prevalence is almost constant in all grid cells but is decreased by 9% compared with H1. For a maximum carrying capacity of *h3* and *h4*, the epidemic peak was respectively 9 and 2 days later than in H1 (Figure 7). Similarly, the local prevalence is decreased by about 2% and 1%, respectively, compared with H1 (Figure 7).

The total prevalence on the grid over time confirms these results. A delay of the epidemic is observed as the density of host-occupied cells decreases. There is also a decrease of the total prevalence compared with H1 (Figure 6).

### Heterogeneous in abundance and distribution of vectors and homogeneous in abundance and distribution of hosts (H3, H4)

#### Hypothesis 3: definition of four subareas

**Figure 8:**
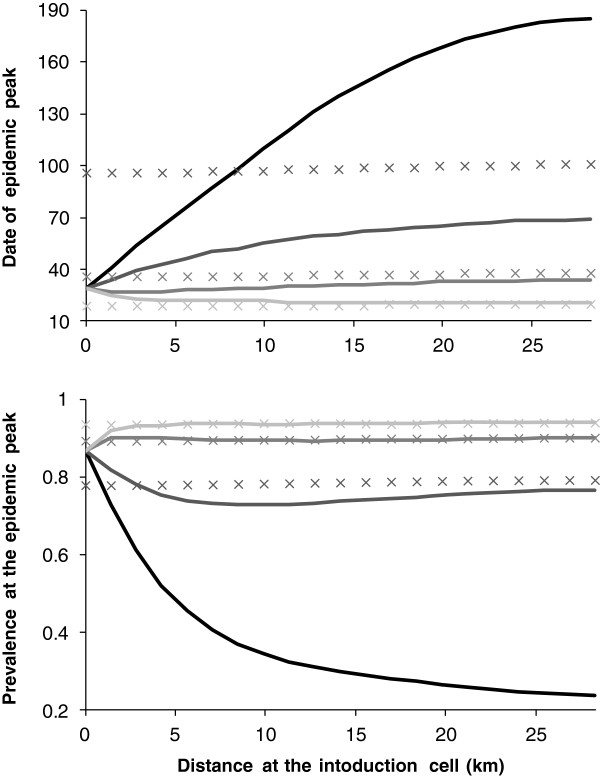
**Peak date and prevalence as a function of the distance from the primary case and the vector maximum carrying capacity.** When hypothesis H3 holds (to help for the comparison, crosses correspond to the homogeneous case, hypothesis H1). *B*lack lines correspond to a maximum carrying capacity in vectors given by *h1* = 10^6^, very dark grey lines correspond to a maximum carrying capacity in vectors given by *h2* = 10^7^, dark grey lines correspond to a maximum carrying capacity in vectors given by *h3* = 10^8^ and light grey lines to a maximum carrying capacity in vectors given by *h4* = 10^9^.

Compared with H1, an epidemic peak is observed in all grid cells, even for the lowest maximum of carrying capacity, *h1* (Figure 8). The further the distance from the grid centre, the more the epidemic peak is delayed and the more the local prevalence decreases. For the carrying capacity *h2*, the observed epidemic peaks are earlier than for H1, including in the cell at the end of the half-diagonal. The local prevalence slightly decreases with increasing distance from the grid centre, to reach an equilibrium equal to the observed infection prevalence at the epidemic peak for H1 (Figure 8). For the two largest maxima of carrying capacity in vectors, *h3* and *h4*, the peak dates and the local prevalences are similar to H1 results (Figure 8). This hypothesis highlights the influence of diffusion in each subarea on the diffusion in others.

#### Hypothesis 4: variable maximum of carrying capacities in vectors

**Figure 9:**
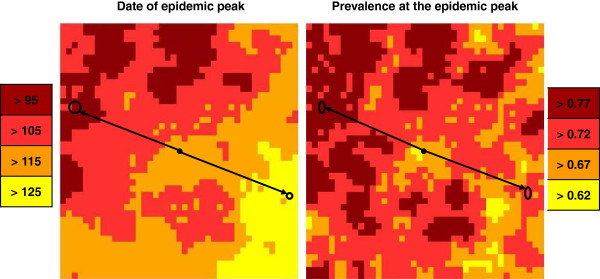
**Spatial distribution of peak dates and prevalence.** When hypothesis H4 holds. The black cell corresponds to the cell wherein the primary case occurs; rounds correspond to cells having the same maximum carrying capacity. From yellow to brown: earlier peak date and increasing prevalence.

Compared with H1, an epidemic peak is observed in all grid cells, even for the lowest maximum of carrying capacity, *h1* (Figure 9). However, the peak dates are delayed. Indeed, epidemic peaks in cells having the same maximum of carrying capacity and equidistant ranged between 96 and 134 days after virus introduction, with no effect of distance on the peak date, (Figure 3, Figure 9). Therefore, cells near the introduction cell can be infected later than cells more distant (Figure 9). While the distribution of the maximum of carrying capacity in vectors is random, clusters of peak dates and of local prevalence arise (Figure 9). The local prevalences vary between 63% and 82% whatever the maximum of the carrying capacity and the distance from the introduction cell (Figure 9). A balance is observed between the peak dates and the local prevalences (Figure 9).

**Figure 10:**
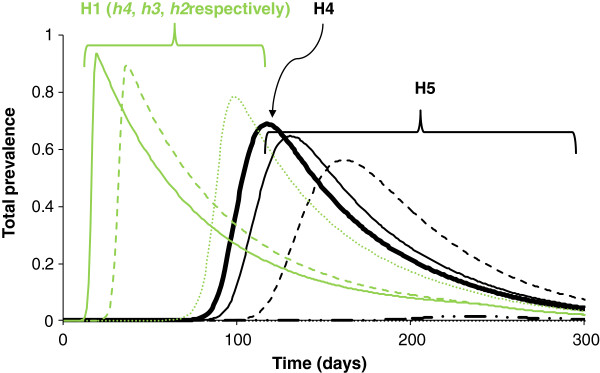
**Total prevalence over time.** In green results when hypothesis H1 holds (same curves as in Figure 6, given here to help for comparison among hypotheses); solid lines correspond to a maximum carrying capacity in vectors given by *h4* = 10^9^, dashed lines correspond to a maximum carrying capacity in vectors given by *h3* = 10^8^, dotted lines correspond to a maximum carrying capacity in vectors given by *h2* = 10^7^. In thick black results when hypothesis H4 holds, in black results when hypothesis H5 holds; solid lines correspond to the case where 90% of cells are occupied, dashed lines correspond to the case where 75% of cells are occupied, dashed-dotted lines correspond to the case where 50% of cells are occupied and dotted lines correspond to the case where 25% of cells are occupied.

The total prevalence on the grid over time confirms these results. A delay of the epidemic and a decrease in the total prevalence are observed compared with H1 (Figure 10).

### Heterogeneous in abundance and distribution of hosts and vectors (H5, H6)

#### Hypothesis 5: theoretical landscape

**Figure 11:**
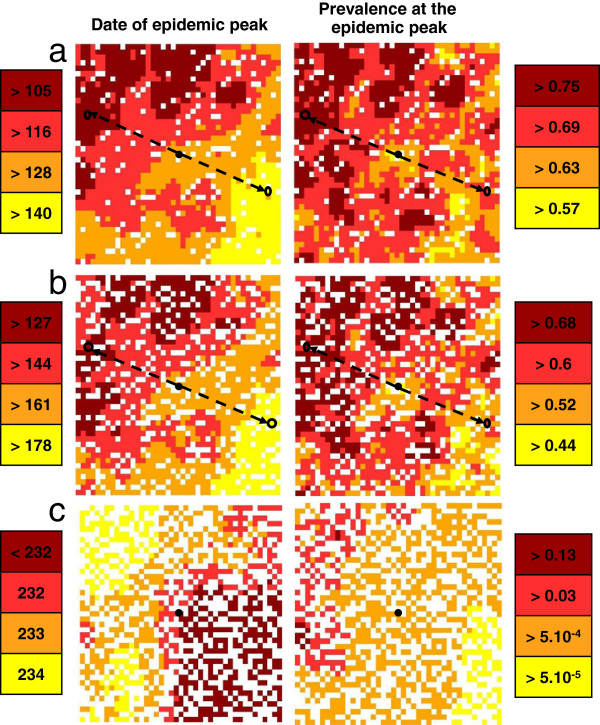
**Spatial distribution of peak dates and prevalence.** When hypothesis H5 holds. **a**) 90% of occupied cells, **b**) 75% of occupied cells, **c**) 50% of occupied cells. The black cell corresponds to the cell wherein the primary case occurs; rounds correspond to cells having the same maximum carrying capacity. From yellow to brown: earlier peak date and increasing prevalence.

As for H2, although later, peak dates are delayed as the density of host-occupied cells decreases (Figure 11). Likewise, the local prevalence decreases when the density of occupied cells in hosts decreases. However, for a density of occupied cells equal to 25%, there is no epidemic; and for a density of occupied cells equal to 50%, the epidemic is small. By comparison with H2 and H4, the same tendencies are observed, with a delay in peak dates and a decrease in local prevalences. The distance has no effect on the peak date, unlike the maximum of carrying capacity in vectors. By comparing the distribution of the maximum of carrying capacity in vectors (Figure 3) with the peak dates and the local prevalences (Figure 11), a balance is observed. Cells with the highest maximum of carrying capacity have an earlier peak date and a larger local prevalence, and vice versa for cells with the lowest maximum of carrying capacity. This is true whatever the distance to the primary case.

The total prevalence on the grid over time confirms these results. A delay in the epidemic is observed as the density of host-occupied cells decreases, as well as a decrease in the total prevalence compared with H1 (Figure 7, Figure 10). A balance is observed between H2-H5 and H4-H5. As for H2 and for a maximum of carrying capacity in vectors of *h2*, the lower the density of host-occupied cells, the longer the peak date is delayed and the lower is the total prevalence (Figure 7, Figure 10). However, the total prevalence for the highest density of occupied cells is similar with hypothesis H4 (Figure 10). Therefore, as soon as the distribution and the abundance of vectors are heterogeneous, they strongly influence the global epidemic dynamics.

#### Hypothesis 6: application to a real landscape

**Figure 12:**
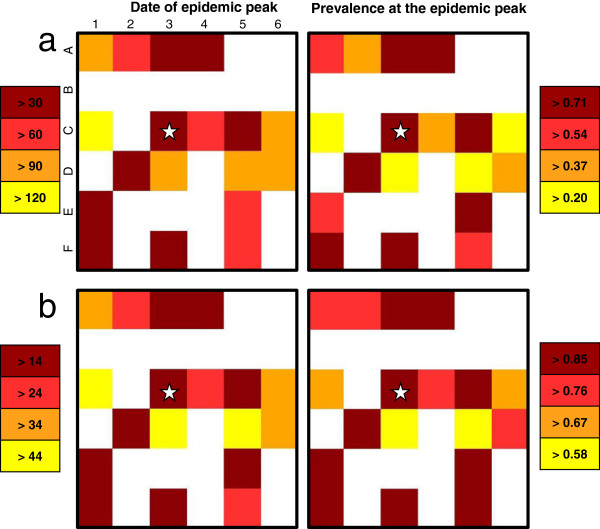
**Spatial distribution of peak dates and prevalence in a real area.** When hypothesis H6 holds. Multiplication of the number of trapped vectors by: **a**) 100; **b**) 1000. The cell with the star corresponds to the cell wherein the primary case occurs. From yellow to brown: earlier peak date and increasing prevalence.

As in the theoretical case, for realistic distribution and abundance of hosts and vectors on a grid, the peak dates and the local prevalences are affected by the maximum of the carrying capacity in vectors in each cell (Figure 12). For roughly equal numbers of hosts and for a high maximum of carrying capacity in vectors (compared to other cells) (cells: A3 and F1, Figure 4), the peak dates and the local prevalences are close (e.g. cells A3 and F1; Figure 12). On the contrary, for roughly equal number of hosts but different maximum of carrying capacity in vectors (e.g. cells C4 and C5; Figure 4), cells with the highest maximum of carrying capacity in vectors have an earlier peak date and a higher local prevalence (e.g. cells C4 and C5; Figure 12). These trends are observed even if the observed number of vectors is multiplied by 100 or 1000, although the peak dates are earlier and the local prevalences higher. Indeed, in our mathematical model, multiplying the observed number of vectors by 100 or 1000 is equivalent to multiplying the maximum carrying capacity of vectors in each cell; that is, to divide *k*_*V*_ by 100 or 1000 in Eq. 1. This has a scaling effect on the model: it does not modify the time periodicity in the population dynamics, but it does change the abundance of vectors. This causes an earlier increase in the number of available infectious vectors and, therefore, the peak dates occur earlier together with higher local prevalences.

In addition, the number of hosts in each cell has an impact on the peak date and the local prevalence. For equidistant cells having the same maximum of the carrying capacity in vectors (e.g. cells A2 and C1; Figure 4a), the cell having the largest number of hosts (C1) shows a later peak date and a lower local prevalence than the cell having the lowest number of hosts (A2; Figure 12).

## Discussion

A mathematical modelling approach allowed us to assess the impact of spatiotemporal heterogeneities in abundance and distribution of hosts and vectors on the spatiotemporal spread of BTV8. Individually and jointly, the heterogeneities in abundance and distribution of hosts and vectors delay the peak date and decrease the total infection prevalence. The different hypotheses of heterogeneity that we have tested allowed us to highlight the importance of the maximum of the carrying capacity in vectors and its influence on the spread of BTV8 within each cell. Indeed, cells next to the primary case can become infected later than more distant cells, if the maximum of carrying capacity is lower. Moreover, the density of cells occupied by hosts plays an important role in cases where the maximum carrying capacity in vectors is low (for homogeneous conditions for vectors) and when hosts and vectors are heterogeneous.

The spatial heterogeneity in host and/or vector abundances influences the infection frequency [7,17,29]. In our study, a decrease in the density of cells occupied by hosts results in a delay of the peak date and a decrease in the infection prevalence if vectors are homogeneously distributed and their population is large. In the case where hosts are homogeneously distributed, the same trend is observed for cells where the maximum of carrying capacity in vectors is large. However, for cells where this maximum is the lowest, an epidemic can occur, in contrast to the case with homogeneous vector and host populations. The coupling of heterogeneities in hosts and vectors increases the delay of the epidemic and decreases the prevalence.

Different models of the spatial spread of bluetongue have previously been published for specific geographic areas [21-24]. Szmaragd’s model describes the BTV spread within and between farms in Great Britain via a generic kernel, which includes both animal and vector movements. Ducheyne’s model was calibrated with data from the BTV1 and BTV8 epidemics in Southern France. It describes the spatiotemporal BTV spread between farms assuming that the number of new cases per week is half attributable to local dispersion (active) of vectors, and half to long-distance dispersion (passive) of vectors by the wind. Graesboll’s model describes the BTV spread with a high spatial resolution, which includes both animal and vector movements and the seasonality of vectors. Turner’s model is a network model. It takes into account explicitly the spatial dispersal of both hosts and vectors and a seasonal vector to host ratio [24]. It studies the BTV spread between farms in England. Taking into account climatic and environmental data, all of these models consider the spatial heterogeneity of the landscape. Our model advances the field by representing spatial heterogeneity in both hosts and vectors. Here, we highlighted how such heterogeneities concretely impact BTV spread. Moreover, the seasonality of the vector population is managed by a simple function that can be easily related to observed data of vector abundance.

We chose to model the spatiotemporal BTV8 spread by a reaction–diffusion model. Several shapes of the transmission kernel are possible, but it is difficult to choose the most appropriate one to describe the dynamics of a pathogen spread. Indeed, Szmaragd et al. showed that a Gaussian kernel was the most appropriate to describe the BTV8 spread in northern Europe during 2006 [21]. If this kernel shape, comparable to reaction diffusion models, has been shown to underestimate the probability of the long-distance transmission, and thus is not appropriate to describe the pathogen spread on a larger scale [30], it can be used on a smaller scale. Graesboll et al. used a Gaussian kernel too, but coupled this approach with the wind dispersion [23]. In our study, the theoretical spatial domain is a 41 × 41 km square. The primary case is always located in the centre of the square, i.e. at 29 km from the domain edges. One limitation is that long-distance dispersal has been neglected; the wind dispersal responsible for the passive movements of vectors generating dispersal up to several hundred kilometres [31,32]. Coupling short and long-distance dispersal is necessary to study arbovirus spread in animal populations once the spatial scale is large enough that host movements and passive movements of vectors cannot be neglected anymore [33-35].

Observational studies have been conducted to assess risk areas and to predict the spatiotemporal spread of BTV in BTV-free areas [11,36,37]. These studies have shown that the landscape heterogeneity, climatic conditions, the distribution and the density of host populations and the abundance of vectors were linked and influenced BTV spatiotemporal spread. Our model highlights similar findings but also allows us to distinguish between the impact of vector versus host heterogeneity. Maps of the basic reproduction number have highlighted the link between vector abundance and BTV spread [12]. However, the vector abundance is difficult to quantify. Hartemink et al. have used trapping data, multiplying the number of trapped *Culicoides* by 100 to obtain a local density of vectors [12]. Our real geographic area illustrates these differences in local abundance. On a small scale, large differences may exist between cells, whether they are occupied by hosts or not. Entomological studies identify and quantify the different vector species present in different geographic locations [9,10,38]. However, the real number of vectors remains difficult to approximate. As shown in our results, the abundance in vectors has a significant impact on the date and on the observed prevalence at the epidemic peak. However, by multiplying the number of vectors by 100 or 1000, we obtained the same qualitative findings on the real geographic area.

Modelling is a relevant approach to investigate the concept of spatiotemporal heterogeneities on the dynamics of virus spread. The distribution of hosts and vectors, and vector abundance strongly influence the dynamics of BTV spread. The application of our model on a real geographic area, although of limited size, allowed us to illustrate the conclusions drawn from a theoretical domain. The reaction–diffusion models are classically used in plant epidemiology [39-41], with the modelled movements generally being for highly volatile entities, and the short *versus* long-distance movements being taken into account via different diffusion coefficients [40,42]. Although developed to represent BTV8 spatiotemporal spread at a local to regional scale, our model can be used to study other vector-borne diseases of animals and its extension to a larger area remains possible.

## Competing interests

The authors declare that they have no competing interests.

## Authors’ contributions

MC and PE conceived of the study, carried out the model development and analysis, and drafted the manuscript. ML and HS conceived of the study, participated in its design and coordination, and drafted the manuscript. MB and GK provided data used in this research, and both commented on draft and final manuscripts. All authors read and approved the final manuscript.
